# Recent advances in mesenchymal stem cell therapy for multiple sclerosis: clinical applications and challenges

**DOI:** 10.3389/fcell.2025.1517369

**Published:** 2025-02-03

**Authors:** Kamran Sheikhi, Salah Ghaderi, Hassan Firouzi, Sarvenaz Rahimibarghani, Ehsan Shabani, Hamed Afkhami, Aref Yarahmadi

**Affiliations:** ^1^ Kurdistan University of Medical Sciences, Kurdistan, Iran; ^2^ Tabriz University of Medical Sciences, Tabriz, Iran; ^3^ Department of Medical Laboratory, Faculty of Medicine, Sari Branch, Islamic Azad University, Sari, Iran; ^4^ Department of Physical Medicine and Rehabilitation, Tehran University of Medical Sciences, Tehran, Iran; ^5^ Department of Clinical Pharmacy, Faculty of Pharmacy, Tehran University of Medical Sciences, Tehran, Iran; ^6^ Cellular and Molecular Research Center, Qom University of Medical Sciences, Qom, Iran; ^7^ Nervous System Stem Cells Research Center, Semnan University of Medical Sciences, Semnan, Iran; ^8^ Department of Medical Microbiology, Faculty of Medicine, Shahed University, Tehran, Iran; ^9^ Department of Biology, Khorramabad Branch, Islamic Azad University, Khorramabad, Iran

**Keywords:** multiple sclerosis (MS), mesenchymal stem cell (MSC), immunomodulation, autoimmune disease (AD), cell therapy

## Abstract

Multiple sclerosis (MS), a chronic autoimmune disorder of the central nervous system (CNS), is characterized by inflammation, demyelination, and neurodegeneration, leading to diverse clinical manifestations such as fatigue, sensory impairment, and cognitive dysfunction. Current pharmacological treatments primarily target immune modulation but fail to arrest disease progression or entirely reverse CNS damage. Mesenchymal stem cell (MSC) therapy offers a promising alternative, leveraging its immunomodulatory, neuroprotective, and regenerative capabilities. This review provides an in-depth analysis of MSC mechanisms of action, including immune system regulation, promotion of remyelination, and neuroregeneration. It examines preclinical studies and clinical trials evaluating the efficacy, safety, and limitations of MSC therapy in various MS phenotypes. Special attention is given to challenges such as delivery routes, dosing regimens, and integrating MSCs with conventional therapies. By highlighting advancements and ongoing challenges, this review underscores the potential of MSCs to revolutionize MS treatment, paving the way for personalized and combinatory therapeutic approaches.

## Graphical Abstract

**Figure:**
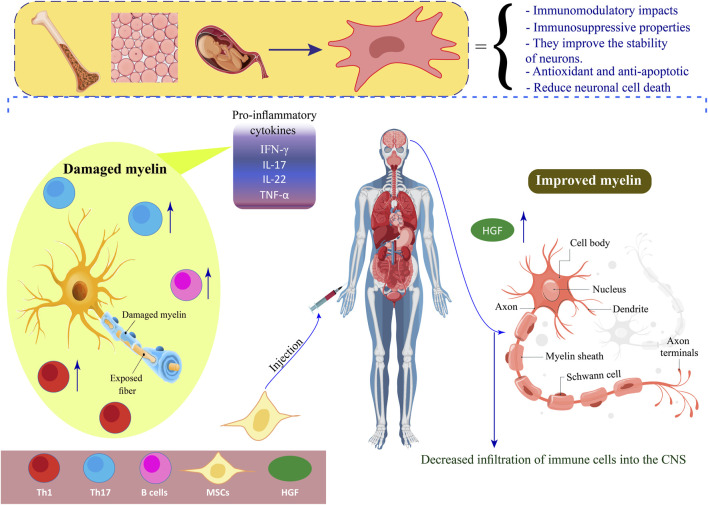
Positive effects of MSCs on MS.

## 1 Introduction

The chronic autoimmune illness of the central nervous system (CNS), known as multiple sclerosis (MS), affects a sizable portion of the global population and has had a profound effect on public health globally (Kobelt et al., 2017; Sumowski et al., 2018; Bjornevik et al., 2022). This illness is typified by inflammation and myelin loss, which results in neurodegeneration. Clinical characteristics include exhaustion and mental/cognitive impairment, in addition to more unusual ones such as vision loss and sensorimotor complaints. It primarily affects female patients and younger people (Penesová et al., 2018). The illness is categorized into three clinical forms: primary progressive (PPMS), secondary progressive (SPMS), and relapsing-remitting (RRMS). Each type is distinguished by a different level of pathology, spanning acute/chronic inflammation and, or neurodegeneration (Stoiloudis et al., 2022). Numerous environmental, dietary, viral (such as the Epstein-Barr virus), genetic, and epigenetic factors may be causal in the onset and progression of MS. The pathophysiology and etiology of MS are complicated (Bjornevik et al., 2022; Ascherio, 2013; Miclea et al., 2020). The MS Atlas estimated in 2020 that one person with MS is diagnosed every 5 min throughout the world, with an average age of 32 years, adding to the 2.8 million people who already have the condition (Charabati et al., 2023). Its frequency varies by region, with Europe and North America having the highest rates (Thompson et al., 2018). Jean-Martin Charcot termed this condition sclérose en plaques in 1868, which was eventually shortened to MS. Charcot and colleagues (Charcot, 1868) discovered that pathological indicators of MS entail the identification of lesions in the regions of the CNS that involve both white and gray matter. These lesions exhibit different levels of demyelination, perivascular immune cell infiltration, reactive gliosis, and, or neurodegeneration. Subsequent research identified abnormalities of the blood-brain barrier (BBB) and axonal transection as additional characteristics of these lesions (Schreiner et al., 2022; Mey et al., 2023). Up till now, several treatment strategies, including fingolimod (FTY720), natalizumab, glatiramer acetate, and interferon-β (IFN-β), have been proposed to regulate aberrant immune responses in MS patients. These medications primarily work by inhibiting immunological responses, which lowers the frequency of relapses and slows the advancement of neurologic impairment. Nevertheless, they have not achieved consistent success (Yousefi et al., 2019; Bejargafshe et al., 2019). According to reports, these therapies are unable to stop the deterioration of nerve tissue in patients with a severe type of MS (Bejargafshe et al., 2019).

**FIGURE 1:**
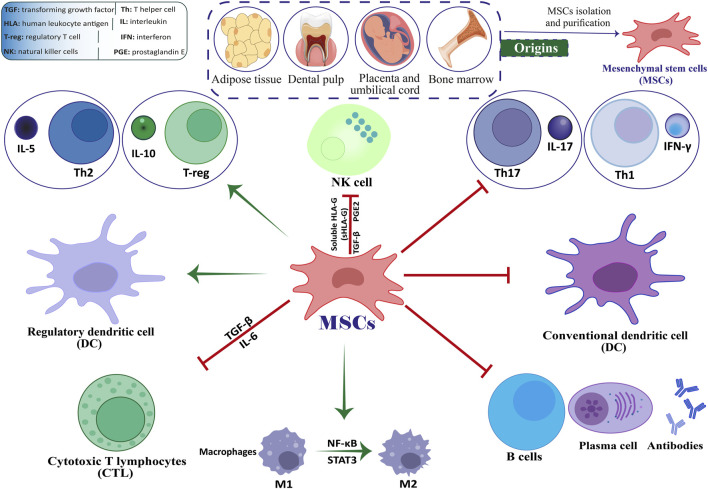
The interplay between MSCs and immune cells.

Stem cell-based therapies, among the various available methods, hold significant potential to effectively reduce neuronal damage in both *in vivo* and *in vitro* models of neurological disorders (Abdallah et al., 2019). The use of mesenchymal stem cells (MSCs) for treating MS has demonstrated encouraging results (ArefNezhad et al., 2023; Zolfaghari Baghbadorani et al., 2023; González et al., 2022; Zhang Y. et al., 2023). Friedenstein and associates identified MSCs as multipotent stem cells in the late 1960s (Friedenstein et al., 1966). Kaplan first used the term “MSCs” in 1991 following his study on human bone marrow (BM) (Caplan, 1991). The capacity of engineered stem cells to multiply (self-renew) and differentiate is well-known. Mammalian tissues such as BM, adipose tissue (AT), dental pulp, amniotic fluid (AF), umbilical cord (UC), etc., are practically all known to contain MSCs. When organs and tissues are damaged, they are in charge of tissue regeneration and repair (Andrzejewska et al., 2019; Yu et al., 2014; Shi et al., 2018). By producing co-stimulatory molecules, they exhibit immunomodulatory features that enable them to control immunological responses and cytokine release (Jiang and Xu, 2020). MSCs are readily extracted from BM, AT, peripheral blood, the placenta, and the UC (Figure 1) (Caplan and Correa, 2011; Laroye et al., 2020). Afterward, they can be grown into a massive population in a culture medium to facilitate cell-based treatment (Planchon et al., 2018). Recently, stem cell-based therapy has given MS patients hope and is currently seen as the most popular noninvasive way to treat many disorders (Xiao et al., 2015).

The primary aim of this review is to investigate and evaluate the valuable capacity of MSCs in managing MS. This includes examining their immunomodulatory and regenerative capacities, discussing findings from preclinical and clinical studies, and identifying challenges such as optimal delivery methods and therapeutic integration. By leveraging the latest advancements in MSC-based research, this review seeks to provide a comprehensive perspective on their clinical applications, limitations, and future directions in MS therapy.

## 2 Pathophysiology of MS

MS, a condition characterized by the breakdown of myelin and loss of axons, is the most frequently encountered non-traumatic debilitating ailment (Hauser et al., 2020). Sclerotic plaques and lesion development in the CNS and cerebrospinal cord are common characteristics of MS (Oh et al., 2018; Kurtzke, 1983). Through controlling synaptic architecture, neurogenesis, and oligodendrogenesis, the immune system plays a crucial role in the evolution of the nervous system. As a result, immune cells may play a part in the cause and development of MS (Rahmati et al., 2021; Sedaghat et al., 2019). Environmental, genetic, and hormonal variables have a significant role in the etiology of MS. Changes in the expression and functionality of immunological agents, including T-cell receptor (TCR), immunoglobulin (Ig), major histocompatibility complex (MHC), and cytokines, have been linked to an elevated risk of MS. According to current MS research, the BBB breach and the start of an autoimmune cascade trigger autoreactive T-cell migration to the CNS, which destroys the myelin sheath and results in sclerotic lesions and plaques. One of the leading causes of MS is the destruction of the myelin sheath, which is essential for axon survival and integration (Khan et al., 2024; Kuhlmann et al., 2023).

MS primarily presents in three distinct clinical courses. RRMS, the most common form of the disease, is characterized by exacerbations followed by complete or partial remissions, affecting 85%–90% of MS patients. After several years, approximately 50%–60% of these individuals progress to SPMS, marked by a gradual worsening of symptoms without remission. Approximately 15% of individuals are diagnosed with PPMS, a condition characterized by a gradual decline in neurological function, with or without episodes of exacerbation (Oliveira et al., 2020).

The primary effector cells involved in the demyelination and destruction of the CNS are T helper 1 (Th1) and T helper 17 (Th17) cells. Specific pro-inflammatory cytokines, such as interferon-gamma (IFN-γ), interleukin-17 (IL-17), tumor necrosis factor-alpha (TNF-α), and IL-1, are produced by Th1 and Th17. Additionally, MS lesions contain CD8^+^ T lymphocytes, particularly in the vicinity of the blood vessels. Prior research has demonstrated that in MS patients, CD8^+^ T cells proliferate more than CD4^+^ T cells, which is mainly linked to axon damage (Khan et al., 2024; Dong et al., 2021; Dadfar et al., 2024). Other immune cells have significant involvement in the development of lesions and plaques in addition to T cells’ responsibilities in the pathogenesis of MS. More CNS antigens are exposed due to myelin being destroyed by Th1 cytokines, which activate macrophages. Although autoreactive T cells are the primary effector cells involved in the development of MS, there have been indications in some studies that autoreactive B cells also contribute significantly to the demyelination and axonal damage by presenting antigens, producing autoantibodies and secreting cytokines (Sedaghat, 2018). Autoantibodies are significant immune mediators present in MS plaques. Several reports suggest a potential correlation between immunoglobulin G (IgG) and the manifestation of symptoms related to MS. Additionally, it has been demonstrated that IgG, particularly IgG targeting myelin fundamental proteins (MBP) and proteolipid proteins (PLP), may be regarded as characteristic markers of the disease. However, their specific roles in the pathogenesis of MS have not been fully elucidated (Maroto-García, 2023; Amin and Hersh, 2023). Research has shown that the introduction of T-cell lines or clones targeting CND myelin antigens into genetically identical, naïve recipient mice has led to the development of experimental autoimmune encephalomyelitis (EAE) as a suitable animal model for MS (Ben-Nun and Lando, 1983; Ben‐Nun et al., 1981; Zamvil et al., 1985). Therefore, inflammation has been seen in MS due to damage to myelin along with axons and neurons, finally resulting in neurodegeneration. The primary means of diagnosis, in addition to clinical presentation, is the temporal and regional appearance of inflammatory lesions as shown by magnetic resonance imaging (Dadfar et al., 2024; Ananthavarathan et al., 2024).

## 3 The mechanisms of action of MSCs in MS

MSCs are multipotent stromal cells that can undergo self-renewal and differentiate into various mesenchymal cell lineages (Dominici et al., 2006; Cesarz and Tamama, 2016; Afkhami et al., 2023; Mirshekar et al., 2023). They can also reduce excessive immune responses and hyperinflammatory processes by inducing the expression of Foxp3+ in CD4 T cells in a laboratory setting (English et al., 2009; Aliniay-Sharafshadehi et al., 2024). MSCs have various ways of regulating the immune system, such as promoting the production of regulatory T cells (Tregs) through direct interaction with T cells and releasing anti-inflammatory substances in a laboratory setting. These mechanisms enable MSCs to manage the development of autoimmune conditions like MS (Figure 1) (Yang et al., 2023; Andalib et al., 2023; Teymouri et al., 2024). When given the right triggers, MSCs can develop into various specialized cell types that originate from mesenchymal tissue, such as bone cells, muscle cells, ligament cells, cartilage cells, and tendon cells (Heldman et al., 2014; Liu et al., 2009; Fakouri et al., 2024). Various non-mesodermal cell lineages have been observed to undergo differentiation, including alveolar cells, hepatocytes, epithelial cells, astrocytes, mature neurons, and neural precursors. These findings indicate that MSCs may play a possible role in the inherent healing process of tissues (Liu et al., 2009; Weiss and Dahlke, 2019; Uccelli et al., 2008).

Previous studies have provided evidence that suggests these cells hold potential as viable treatment options for a range of neurological disorders, such as MS and amyotrophic lateral sclerosis (ALS) (Najafi et al., 2023; Vaheb, 2024). The immunomodulatory impacts of MSCs may be demonstrated through their direct engagement with immune cells or through the transmission of paracrine signals. Research has shown that MSCs can inhibit the differentiation of Th17 and Th1 cells. Research has indicated that MSCs expanded in a laboratory setting can hinder the growth of T lymphocytes, B lymphocytes, and natural killer (NK) cells, as well as impede the maturation and differentiation of dendritic cells (DCs) (Yang et al., 2023; Mei et al., 2024). In recent times, stem cell-based therapy has become a promising strategy for treating patients with MS. It is currently considered the most preferred and least intrusive treatment option for a range of medical conditions (Papaccio et al., 2017; Islam et al., 2023).

In RRMS, inflammation is dominant, driven by autoreactive T cells and a disrupted BBB. Several studies have demonstrated a reduction in relapse frequency and lesion formation in preclinical models of RRMS following MSC therapy (Vaheb, 2024; Gavasso et al., 2024). Applying MSCs in clinical research involving patients with RRMS has yielded promising results. The study observed a trend toward reduced levels of pathogenic inflammatory Th1 and Th17 cell subtypes, accompanied by a decrease in inflammation as indicated by MRI scans. Notably, there was also an increase in regulatory B cells (Llufriu et al., 2014a). In progressive forms of MS, such as PPMS and SPMS, the primary challenge lies in addressing neurodegeneration and promoting repair mechanisms. To treat PPMS and SPMS, MSC therapy has been investigated as a potential option for targeting various therapeutic targets (Gavasso et al., 2024; Ghareghani et al., 2024). The first placebo-controlled trial utilizing intrathecal administration of MSCs in patients with active progressive MS demonstrated a positive impact on disease outcomes. This included reduced neurofilament light chain levels (NfLs), stabilization or improvement of disability scores, and the achievement of status without evidence of disease activity (Roig-Carles et al., 2021; Colasanti et al., 2014). Despite showing signs of neuroprotection, MSC did not seem to influence humoral immunity to common antigens or peripheral T-cell subsets in the context of SPMS (Connick et al., 2012). This suggests that there may be significant differences in the mechanisms underlying the effects of MSCs in RRMS and progressive MS. It highlights the idea that, depending on the local environment, disease state, and phenotype, MSCs exhibit diverse immunomodulatory effects on various types of immune cells (Zhao, 2019).

MSCs have the potential to provide structural support to axons and improve the stability of neurons. Additionally, they are believed to possess antioxidant and anti-apoptotic properties and can release trophic factors. Additionally, they have the potential to facilitate the generation of fresh neurons and glial cells, such as oligodendrocytes (ODCs) (Guimarães-Camboa et al., 2017; Song et al., 2018). In individuals with MS, MSC has the potential to augment the differentiation of neural cells, reduce neuronal cell death, and stimulate the formation of new blood vessels, ultimately contributing to the repair of the CNS (Gavasso et al., 2024). According to recent research, it has been determined that MSCs can enhance peripheral tolerance by suppressing the differentiation and function of DCs, consequently diminishing antigen presentation and impeding the expansion of self-reactive T cells (Zhuo et al., 2023). In addition, MSCs can produce hepatocyte growth factor (HGF), which leads to an increase in tolerogenic DCs. Research has revealed that the administration of MSCs in combination with HGF results in a reduction of CNS inflammatory reactions and the infiltration of immune cells in mice with EAE. Consequently, MSCs derived from HGF demonstrate potential as a viable therapeutic approach for MS and other autoimmune disorders (Bai et al., 2012; Mansoor et al., 2019).

IL-6 and CD20 are key molecules intricately linked to the inflammasome and immune regulation, playing significant roles in the inflammatory cascade observed in MS (Margoni et al., 2022; Chmielewska and Szyndler, 2023). One of the most well-known pro-inflammatory cytokines is thought to be IL-6. More than 100 nations have approved using the neutralizing monoclonal antibody tocilizumab to treat autoimmune diseases by blocking IL-6 (Kishimoto, 2005; Tanaka, 2014). While circulating IL-6 levels are as low as 1–5 pg/mL under homeostatic settings, they may increase by over 1,000 times during inflammatory states, and in severe situations that result in sepsis, IL-6 levels as high as µg/mL have been seen (Waage et al., 1989). IL-6 is synthesized by myeloid cells in response to stimulation of Toll-like receptors, in conjunction with the cytokines IL-1β and TNF-α. This interaction initiates a feed-forward loop that significantly enhances the production of IL-6 in the context of inflammatory responses (Tanaka et al., 2014). IL-6 is a key mediator in the activation of the inflammasome, playing a significant role in chronic inflammation and tissue damage associated with MS. Elevated levels of IL-6 have been correlated with disease severity, underscoring its contribution to the persistence of neuroinflammation and the impairment of remyelination processes (Stampanoni Bassi et al., 2020; Vandebergh et al., 2022).

MSCs possess the ability to express and secrete various cytokines, including IL-6. However, they generally produce lower levels of IL-6 than immune cells such as T cells and macrophages. Under certain conditions, such as exposure to inflammatory stimuli or interaction with immune cells, MSCs can increase their production of IL-6 (Kerkis et al., 2024; Philipp et al., 2018; Huang et al., 2022). MSCs demonstrate potential in regulating IL-6 expression in the context of neuroinflammation. Both autocrine and paracrine signaling loops, along with feedback control from the immune system, contribute to the downregulation of IL-6 by MSCs (Hofer and Tuan, 2016; Lopez-Santalla et al., 2020; Molnar et al., 2022). The complex relationship between MSCs and endogenous IL-6 production depends on experimental conditions and cellular interactions (Dorronsoro et al., 2020). Gu et al. (2016) demonstrated that the release of endogenous IL-6 induced by MSCs led to an upregulation of IL-6 receptor (IL-6R) and phosphorylated signal transducer and activator of transcription 3 (p-STAT3) levels in astrocytes subjected to oxygen and glucose deprivation. Notably, a significant increase in the ratio of B-cell lymphoma 2 (Bcl-2) to Bcl-2-associated X protein (Bax), critical downstream components of the STAT3 signaling pathway, was observed. This study elucidated the neuroprotective effects of MSC transplantation in a rat model of neonatal hypoxic-ischemic brain injury, suggesting that these effects are partially mediated by IL-6, which enhances the anti-apoptotic properties of damaged astrocytes through the IL-6/STAT3 signaling pathway. Through paracrine signaling and immunomodulatory mechanisms, MSCs have demonstrated their ability to inhibit the production of IL-6 by immune cells such as T cells and macrophages. However, MSC-derived IL-6 has also been shown to stimulate or modulate the activity of other immune cells, which in turn affects endogenous IL-6 levels (Song et al., 2020; Toh, 2017; Glenn and Whartenby, 2014). MSCs have anti-inflammatory properties that influence IL-6 levels in a variety of settings. They can also reduce IL-6 synthesis by inhibiting immune cell activation (Dabrowska et al., 2021; Saadh et al., 2023).

CD20, a surface marker predominantly expressed in B cells, is another molecule associated with the pathogenesis of MS. It plays a crucial role in B cell activation, antigen presentation, and the production of pro-inflammatory cytokines (Margoni et al., 2022; de Sèze et al., 2023). In recent years, there has been a growing interest in CD20-targeting therapies, specifically anti-CD20 monoclonal antibodies that facilitate B-cell depletion, including ocrelizumab, rituximab, and ofatumumab. The therapeutic scenario for treating MS patients has significantly expanded due to the remarkable effectiveness and favorable safety profile of these selective B-cell-depleting treatments (Hauser et al., 2020; Hauser et al., 2008; Montalban et al., 2017). MSCs can complement this approach by further modulating B cell activity, promoting regulatory B cells, and inhibiting the production of autoantibodies that exacerbate disease progression (Yordanova et al., 2024; Veh et al., 2024). The negative regulatory influence of MSCs on B lymphocytes may result from direct contact with B cells, leading to the release of various soluble cytokines that impact B cell function. This, in turn, prevents B cells from proliferating and reduces the generation of memory B cells and plasma cells, which decreases the number of B cells that secrete cytokines, chemokines, and antibodies (Hoorweg et al., 2015). MSC can enhance the synthesis of granulocyte-macrophage colony-stimulating factor (GM-CSF) through the involvement of stem cell antigen 1/lymphocyte antigen 6AIE protein while simultaneously inhibiting the maturation of B lymphocytes. Transforming growth factor-beta (TGF-β) secreted by MSCs plays a crucial role in suppressing B lymphocytes by downregulating or inhibiting IL-7 produced by stromal cells (Hoorweg et al., 2015; Liu et al., 2015). The application of MSCs in MS, particularly concerning IL-6 and CD20, highlights their dual role in targeting innate and adaptive immune responses.

### 3.1 Routes of administration, dosing, and infusion vehicles for MSC therapy in MS

The success of MSC therapy in MS is significantly influenced by the route of administration, dosing regimen, and infusion vehicle. Preclinical and clinical studies have primarily utilized intravenous (IV) and intrathecal (IT) routes. IV administration allows systemic delivery, while IT targets the CNS directly, potentially enhancing therapeutic efficacy (Table 1) (Cohen et al., 2018; Iacobaeus et al., 2019; Harris et al., 2016; Harris et al., 2018). Empirical investigations have demonstrated that the IV delivery of MSCs exhibits immunosuppressive properties and mitigates the symptoms of autoimmune disorders (Sato et al., 2009; Jiang et al., 2017). Studies have also shown that the transfer of MSC results in a notable improvement in the clinical results of MS in experimental models of EAE (Bazinet and Popradi, 2019; Alanazi et al., 2022). Recent clinical trials have investigated the effectiveness and safety of MSCs in treating MS. The tests have shown that MSCs, when administered intrathecally into the cerebrospinal fluid (CSF) of the spinal cord, can successfully migrate to brain lesions (Alanazi et al., 2022; de Witte et al., 2018; Uder et al., 2018). This intervention is expected to enhance the viability of brain cells by promoting their transformation into precursor cells for neurons and glial cells, thus mitigating the impairment of brain function. Consequently, this approach can potentially reduce the severity of the disease and enhance the overall wellbeing of individuals affected by MS (Von Wunster, 2018; Neal et al., 2018). Syngeneic MSC via IV administration in the EAE model induces tolerance in myelin ODC glycoprotein (MOG)-specific T cells. This leads to a reduction in immune cell infiltration into the CNS, an amelioration of clinical outcomes, and decreased myelin degradation (Freedman et al., 2010).

**TABLE 1:** Summary of preclinical studies on stem cell therapies in animal models of MS.

Study	Animal	MS model	Type of stem cell	Amount, Route, and time of appl.	Results
Liu et al. (2019)	Cynomolgus monkey	EAE by MOG34-56	UCMSCs	1 × 10^6^ cells/kg/mL, IV, On days 74 and 84	-↓ IL-5, IFN-γ, GM-CSF, and IL-17A
					-↑ IL-8, IL-4 and IL-10
					-Enhances Treg populations and NK cells
					-Suppresses astrocyte activation, decreased proportions of Th1 and Th17 cells
					-↓ Demyelination in MRI
Liu et al. (2020a)	C57BL/6 mice	EAE, MOG33–35	MSC (from the whole spinal cord)	5 × 10^5^, via tail vein, 11 dpi	-Reduced IL-6, IL-1β, IL-12, and TNF-α expression
					-Downregulated AQP4 and A2BAR Expression
					-Reduced neutrophil infiltration
					-Reduced IgG leakage
					-Reduced BBB Disruption
					-Reduced Spinal Cord Demyelination
					-Improved Neurobehavioral Outcomes
					-MSCs demonstrate a positive impact by preserving the integrity of the BBB in mice with EAE
Barkat (2020)	C57BL/6 mice	Cuprizone intake	AD-MSCs	1 × 10^6^, single IV injection	-Decrease the oxidant level
					-Enhanced the remyelination
					-Improved motor and cognitive functions,
Clark et al. (2019)	C57BL/6J mice	EAE, MOG33–35	Placental MSC-derived extracellular vesicles (PMSC-EVs)	1 × 10^7^ PMSC-EVs (low dose),	-PMSCs secrete high levels of BDNF, HGF, and VEGF
				1 × 10^10^ PMSC-EVs (high dose), via tail vein,	-Improved motor function scores (in high dose),
Nasri et al. (2018)	Mice	EAE by MOG34-56	MSC-derived Neural Progenitors (MSCs-NPs)	1 × 10^6^ cells, IV, 22, 29, and 36 dpi	-Significant decrease in IFN-γ or IL-17,
					-Increased IL-10 and PGE2
					-Suppressive immunity system
					-Significantly decreased the clinical scores
					-Unlike MSCs, allogeneic MSCs-NPs demonstrate greater efficacy in mitigating EAE
Mahfouz et al. (2017b)	Swiss mice	EAE, emulsion of rat SCH	BM MSC and MP	1 × 10^6^ cells,	-MPO activity and decreased TNF-α content increased IL-10
				1 × 10^6^/mouse, intraperitoneal (IP), once/14, 28 dpi	-Decrease hemorrhage, edema, and neuronal viability parameter
					-MSCs did not contribute to the alterations observed in amino acids and neurotransmitters following the induction of EAE
Pinheiro et al. (2019)	Dogs	With evident signs of demyelinating leukoencephalitis	Flank AT of each canine patient	Three injection of 1 × 107 MSCs were administered intra-arterially, with a 30-day interval between each infusion	-No significant differences were observed before and after administering the three injections. Additionally, there were no alterations in the participants' laboratory test results before and after 1 year of treatment
Gramlich et al. (2020)	C57BL6/J (B6) mice	EAE induction using MOG35–55	Human BM-derived MSC	10^6^ MSC, via tail vein, 7	-The administration of MSC treatment at a systemic level has a beneficial impact on the function and survival of retinal ganglion cells (RGC) in mice with EAE
					-MSC therapy has been shown to decrease endoplasmic reticulum stress and HIF-1 signaling and to have a positive impact on cholesterol metabolism in the retinas and optic nerves of mice with EAE
Yan et al. (2018)	Cynomolgus monkeys	EAE induction using MOG35–55	Human embryonic stem cells (EMSC)	2 × 10^7^ cells/monkey (IT)	-When administered intrathecally into the CNS, EMSCsp significantly decreased the clinical manifestations, brain abnormalities, and demyelination of neurons in the EAE monkeys over 3 months
				IV injection. Single cells (1 × 10^7^ cells/kg),	-Furthermore, EMSC demonstrated the ability to undergo transdifferentiation into neural cells within the CNS of the treated animals
				19 and 33 dpi	-The direct injection of EMSCsp into the CNS can mitigate the progression of the disease in the primate EAE model, showing promise for potential clinical application
Barati et al. (2019)	C57BL/6 mice	fed by cuprizone	BM-MSC	3 × 10^5^, injected into the right lateral ventricle, 14 weeks	-The findings indicated that trophic factors released by MSC could potentially enhance the population of ODCs and the rate of remyelination by mitigating the presence of pro-inflammatory factors
					-MSCs can potentially reduce inflammation, demyelination, and gliosis through their neuroprotective and immunomodulatory properties in a chronic cuprizone demyelination model
Laso-García et al. (2018)	SJL/J mice	*Ad libitum* access to water and food	Human AT-derived MSCs	IV, 2 × 10^6^, 75 days	-Reduced levels of IL-12p70, IFNγ cytokines Th1, and Th17 in the TMEV-EVs mice
					-The TMEV-VH group exhibited a significantly greater lesions than the TMEV-EVs group
					-Significant reduction in GFAP for astrocytes and Iba-1 for microglia
					-Significant increase in myelin proteins such as CNPase and MBP
Sadeghnejad et al. (2024)	C57BL/6 mice	EAE-induced using MOG	MSCs	IV,----	-Significant reduction in clinical symptoms
					-Decreased lymphocyte infiltration into the spinal cord
					-Reduced demyelinated areas
					-Enhanced production of anti-inflammatory cytokines IL-10, TGF-β, and IL-4
					-Decreased production of pro-inflammatory cytokines IFN-γ and TNF-α
Haghmorad et al. (2023)	C57BL/6 mice	EAE-induced	BM-MSCs	1 × 10^6^ BM-MSCs, Intraperitoneal injection, Administered in three-time schemes:	- Improved cognitive function, as demonstrated by a decreased EAE clinical score
				Day 6 post-EAE induction,	- Decreased inflammation and demyelination in brain tissues
				Days 6 and 12 post-EAE induction,	-Increased miR-193 and miR-146a (anti-inflammatory)
				Day 12 post-EAE induction	-Decreased miR-155, miR-21, and miR-326 (pro-inflammatory)
					- Suppressed Th1/Th17 immune responses with reduced IFN-γ and IL-17 cytokine levels
Perussolo et al. (2024)	Rats	EAE induced by myelin essential protein	Human Wharton’s Jelly MSCs and Neural Precursors	1 × 10^6^ cells, IV injections, administered at disease onset	-Reduced inflammation, improved functional recovery, and enhanced remyelination.

Dosing varies widely, from single infusions of 1 × 10^6^ cells/kg to repeated doses administered monthly or biannually. Liu et al. (2019) administered umbilical cord mesenchymal stem cells (UC-MSCs) IV in Cynomolgus monkeys with EAE on days 74 and 84, using a dose of 1 × 10^6^ cells/kg/mL, which significantly reduced pro-inflammatory cytokines such as IL-5 and IFN-γ while increasing anti-inflammatory cytokines like IL-10. Similarly, MSCs derived from the whole spinal cord were injected into C57BL/6 mice via the tail vein at a dose of 5 × 10^5^ cells 11 days post-immunization (dpi), resulting in reduced inflammation, improved BBB integrity and enhanced neurobehavioral outcomes (Liu Y. et al., 2020). In another study by Clark et al. (2019), placental MSC-derived extracellular vesicles (PMSC-EVs) were infused at doses of 1 × 10^7^ and 1 × 10^10^ PMSC-EVs, showcasing the dose-dependent improvement of motor function scores in EAE-induced mice (Clark et al., 2019).

Moreover, the mode of infusion plays a vital role in the therapeutic efficacy of MSCs. Barati et al. (2019) administered BM-MSCs directly into the right lateral ventricle in a cuprizone-fed demyelination model of mice, emphasizing the localized enhancement of ODC populations and remyelination processes. Yan et al. (2018) demonstrated the importance of IT and IV routes for human embryonic stem cells (EMSCs) in Cynomolgus monkeys. IT injections showed superior outcomes in reducing brain abnormalities and demyelination compared to IV. Importantly, infusion vehicles such as saline or specialized buffers were crucial for cell viability and delivery efficiency, though specific details on vehicles were sparsely reported (Fernández-Santos et al., 2022). Collectively, these studies underscore the significance of optimizing the route, dose, and infusion medium to maximize the immunomodulatory and neuroprotective potential of MSC-based therapies in MS models.

### 3.2 Proliferation of oligodendrocytes (ODCs)

ODCs function as the cells responsible for myelination within the CNS. They are derived from precursor cells of ODCs through intricately coordinated processes involving migration, differentiation, and proliferation (Moore et al., 2020; Zeisel et al., 2015; Bradl and Lassmann, 2010). ODCs play a crucial role in developing myelin in the CNS and are essential for the regenerating myelin after injury, including in the prevalent demyelinating disease MS (Franklin and Ffrench-Constant, 2017; Nave, 2010). The ODCs surrounding axons in the CNS have been crucial in improving the speed of nerve impulse conduction, maintaining the structural integrity of axons, and directly supplying metabolic support to lengthy axons (Thompson et al., 2018; Zhang et al., 2016; Kassmann et al., 2007). The inability to form a myelin sheath, known as myelination, or the breakdown of the myelin sheath due to diseases or injuries, disrupts the efficient transmission of action potentials in the vertebrate nervous system, ultimately contributing to the development of neurodegenerative diseases such as MS (Vincze et al., 2011; Oudejans et al., 2021). MSCs significantly increased the amount and size of ODC processes; moreover, inhibition experiments demonstrated that the soluble factor Sonic hedgehog created by EMSCs, extracellular matrix molecule, and laminin gap junction protein connexin 43 were responsible for stimulating OPC differentiation because preventing the function of either of the three proteins resulted in significant retraction of processes and ODC detachment. The MSC culture system might be a model for enhancing ODC differentiation and maturation. MSCs could be a promising cell resource for treating neurological disorders related to ODC destruction and demyelination (Zhang et al., 2016; Aggarwal and Pittenger, 2005; Rivera et al., 2019; Manu et al., 2021).

## 4 Methods of using MSCs

### 4.1 Naive MSC

MSCs are generally a group of cells that adhere to surfaces and can renew themselves and transform into various cell types, such as bone, fat, and cartilage cells. Furthermore, MSCs demonstrate significant promise in the modulation of the immune system and exhibit low immunogenicity. MSCs derived from various sources exhibit comparable characteristics. These cells hold promise for applications in regenerative medicine and contribute to maintaining tissue equilibrium (Li et al., 2019; Shang et al., 2021; Casado-Díaz et al., 2020; Nethi et al., 2023; Farokhi et al., 2024).

MSCs were observed to migrate to the injured brain, indicating their potential as a promising cell source for regenerating damaged organs, including the CNS (Li et al., 2002; Andrzejewska et al., 2021). Induction of EAE in mice enhanced brain thiobarbituric acid reactive substances (TBARS) and nitric oxide (NO), TNF-α, and myeloperoxidase (MPO) and decreased brain glutathione (GSH) content and IL-10, compared to the control group. MSC therapy reduced NO, TBRS, TNF-α, and MPO levels while increasing GSH and IL-10 range. This suggests that MSC therapy may be a practical approach for reducing oxidative stress and inflammatory responses in the CNS (Mahfouz et al., 2017a; He et al., 2021).

Recently, Li et al. demonstrated that menstrual blood-derived mesenchymal stem cells (MB-MSCs)- and umbilical cord-derived mesenchymal stem cells (UC-MSCs) could ameliorate MS severity when transplanted at different phases of EAE by either IV or intraperitoneal (IP) route. They identified decreased Th1 and Th17 cell response, which, in turn, led to reduced severity of EAE disease. As a result, in MS-related inflammation, they concluded that MSCs could be used as allo-MSCs (Ling et al., 2022).

Furthermore, their minimal immunogenicity, related to a low expression of MHC-I and an absolute lack of MHC-II (Wang et al., 2019; Ankrum et al., 2014) and co-stimulatory molecules, allows them to elude immune surveillance (Han et al., 2019). In another study, MSCs were differentiated into neurotrophic factor-producing cells (NTFCs) *in vitro* to investigate the clinical usage of NTFCs for EAE symptoms. The NTFCs and MSCs were injected intracerebroventricularly (ICV) into EAE mice, resulting in delayed symptom onset and raised animal survival. MSCs and NTFCs were found to suppress mouse immune cells and protect brain cells from oxidative stress (Barhum et al., 2010). Moreover, systemic administration of MSCs enhanced the expression of neural progenitor markers, including nestin (NESTIN), paired box protein Pax-6 (PAX6), vimentin (VIMENTIN), and class III beta-tubulin (TUJ1), in the brains of treated MS rodent models. Analysis revealed that MSCs home the CNS produced an anti-inflammatory mediator, enhanced Treg cell numbers, and induced neuroprotection and myelination in treated models (Brown et al., 2021). Recent reports also demonstrated that co-administration of MSC and FTY720 could exert better therapeutic benefits compared to the administration of each of them. This combination therapy drastically decreased axonal loss and inflammatory CNS infiltrations. Accordingly, FTY720 may promote future immunomodulatory medication and cellular therapy combinations to enhance the advantages of progressive MS (Kassis et al., 2021).

Additional research has demonstrated that the inclusion of rapamycin in bone marrow-derived mesenchymal stem cell (BM-MSC) transplantation in EAE mice resulted in a notable decrease in demyelination and inflammation infiltration, an enhancement of immunomodulatory functions and a suppression of the advancement of neurological impairments when compared to BM-MSC transplantation alone and control groups. BM-MSC and rapamycin co-treatments had immunological effects that increased the production of the IL-4, IL10, and Th-2 cytokines and decreased CD8^+^ cytolytic activity, Ag-specific lymphocyte proliferation, and Th1-type cytokines (Togha et al., 2017; Xin et al., 2020; Ceccariglia et al., 2020). The use of rapamycin with BM-MSCs illustrates the potential of combining immunomodulatory therapies for more effective MS treatment.

Table 1 is an overview of studies investigating the effect of stem cells (especially MSCs) on various animal models of MS.

### 4.2 Primed or pretreated MSCs

Empirical evidence indicates that MSCs derived from various sources and delivered using different techniques can reduce inflammatory cell infiltration and demyelination, resulting in symptom improvement and better clinical outcomes. Moreover, preconditioned or differentiated MSCs, as well as MSCs combined with other compounds, demonstrate greater therapeutic potential and provide enhanced protection in MS models compared to native MSCs (Gugliandolo et al., 2020; Kilian et al., 2010; Brown et al., 2019; Mahjoor et al., 2021; Mahjoor et al., 2023a). Recent reports have delivered proof that estradiol plays an essential role in controlling several MSC functions, including the synthesis of vascular endothelial growth factors (VEGF) and procedures of cell proliferation (Erwin et al., 2009; Mihai et al., 2019; Cho et al., 2021). Meanwhile, MSCs primed with 17β-estradiol (17-ED) exhibited enhanced therapeutic efficacy compared to naïve MSCs in EAE rat models. This was demonstrated by improved neuropathological changes, a reduced total clinical score, and a significant increase in body weight (Heidari barchi nezhad et al., 2018). Besides, tetramethylpyrazine treatment reduced apoptosis in UCMSCs and enhanced their proliferation *in vitro* and *in vivo*. Furthermore, tetramethylpyrazine-UCMSC treatment significantly decreased clinical scores, demyelination, BBB disruption, and inflammation in experimental EAE mice (Zhang et al., 2020). Ling et al. also exhibited that IFN-γ-UC-MSCs transplantation considerably reduced clinical scores and body weight loss of EAE mice more evidently compared to naive UCMSCs. The intervention also reduced IL-17 levels in treated mice, conferring the patent anti-inflammatory role of IFN-γ-UCMSCs *in vivo* (Ling et al., 2022). Similarly, IFN-γ enhanced the secretion of indoleamine 2, 3-dioxygenase 1 (IDO1), a valuable biomolecule produced by MSCs to perform their immunosuppressive function. Meanwhile, it has been suggested that IFN-γ-UCMSCs systemic administration resulted in decreased levels of TNF-α in EAE mice. Likewise, IFN-β- adipose-derived mesenchymal stem cells (ADMSCs) preserved and promoted the functional features in EAE mice primarily by reducing central and peripheral neuroinflammation (Zhou X. et al., 2020; Marin-Bañasco et al., 2017).

In conclusion, priming or preconditioning MSCs with various molecules, such as estradiol or IFN-γ, significantly enhances their therapeutic potential in MS models. These approaches contribute to more effective modulation of immune responses and inflammation, presenting a promising avenue for MSC-based therapies in MS treatment.

### 4.3 Genetically modified MSCs

Genetic modification caused improved migration, adhesion, and survival, preconditioning change, and reduced premature senescence in MSCs. In the process of genetic modification, a newly created gene sequence is inserted into the vector to facilitate its entry into the MSCs. Once inside the MSC, it activates the expression of particular genes or causes them to be overexpressed. A gene switch may be used to modulate transgenic expression, or it may remain constant, resulting in the specific production of particular molecular proteins (Phillips and Tang, 2008; Ocansey et al., 2020; Lan et al., 2020). Different genetic engineering techniques have been used to improve the gene expression patterns of MSCs. These methods can be categorized as those using non-viral or viral vector methods. Replication-deficient viruses, which are commonly used as gene transfer agents, are preferred due to their effective DNA transfer capabilities. However, their clinical use is limited by the high cost of generating cell lines and the potential for immune responses (Park et al., 2015). In contrast, non-viral methods, which include physical or chemical processes, are less immunogenic and can be produced in large quantities. Physical methods for genetically modifying MSCs include nucleofection, sonoporation, and electroporation, while chemical techniques employ lipidic molecules, inorganic nanoparticles, and polymers (Damasceno et al., 2020).

Recent *in vivo* studies have demonstrated that MSCs genetically engineered to produce IL-4, a cytokine known for modulating the autoimmune inflammatory response, exhibited enhanced protective effects when transplanted during the early stages of the disease. Compared to unmodified MSCs, MSC-IL-4 significantly reduced the production of pro-inflammatory cytokines such as IFN-γ and IL-6, leading to a decrease in disease severity (Payne et al., 2012). Rostami et al. also found that IL-23 receptor (RIL-23R) mRNA transfection significantly improved MSC features in the inflamed areas of EAE models and increased their ability to control the proliferation of T lymphocytes. MSCs-IL-23R also showed a more substantial therapeutic effect than MSCs during *in vivo* therapy in EAE mice, as documented with increased myelination and a decrease in the entrance of inflammatory mediators into the white matter (Rostami et al., 2022). Moreover, transfecting MSCs with P-selectin glycoprotein ligand-1 (PSGL-1) and sialyl Lewis X (SLeX) mRNA significantly enhanced MSC homing to inflamed areas *in vivo*. The overexpression of PSGL-1/SLeX increased the rolling and adhesion of cells on brain microvascular endothelial cells and contributed to the integrity of the BBB in EAE mice (Liao et al., 2016). In another study, CD4^+^ T cell proliferation isolated from EAE mice was significantly inhibited by MSCs modified to overexpress IL-10. Wang et al. also demonstrated that transplanting sphingosine kinase 1 (SPK1) gene-modified UC-MSCs (UCMSC-SPK1) significantly decreased the intensity of the neurological impairment in EAE mice models by reducing axonal loss, demyelination, and astrogliosis. Additionally, UCMSC-SPK1 transplantation upregulated the proportion of FoxP3+ (Treg) CD4^+^ CD25^+^ T cells and facilitated the development of NK cell responses in the EAE mice’s spleen (Wang et al., 2018). In another study, researchers used MSCs as a treatment plan and a vehicle to transfer fully processing 3.3-kDa vasoactive intestinal peptide (VIP) to the inflamed CNS and peripheral immune organs. Intraperitoneal injection of MSCs-VIP reduced neuroinflammation and demyelination and increased CNS neuronal integrity in part by inhibiting T-cell activation (Cobo et al., 2013).

In conclusion, genetic modification of MSCs offers a promising strategy to enhance their therapeutic efficacy in MS models. By modifying MSCs to produce specific cytokines or surface molecules, their migratory, immunomodulatory, and neuroprotective properties are significantly improved, providing a potential avenue for more effective MS treatments (Rostami et al., 2022; Li et al., 2022; Moeinabadi-Bidgoli et al., 2023).

### 4.4 Introduction to the MSC secretome

MSCs possess a secretome that comprises both a soluble and a vesicular fraction. The soluble fraction contains numerous neurotrophic growth factors, chemokines, and cytokines, including IL-6, IL-10, IL-17, prostaglandin E2 (PGE2), C-X-C motif chemokine ligand 10 (CXCL-10), glial-derived neurotrophic factor (GDNF), brain-derived neurotrophic factor (BDNF), VEGF, fibroblast growth factor (FGF), HGF, nerve growth factor (NGF), and insulin-like growth factors 1 and 2 (IGF-1 and IGF-2). The vesicular fraction contains extracellular vesicles (EVs) of various sizes, including exosomes (Pinho et al., 2020; Kumar et al., 2019; Mahjoor et al., 2023b). Through T-cell inhibition and macrophage regulation, studies have shown that the secretome of MSCs lowers inflammation. This leads to decreased pro-inflammatory cytokine production and better results in mouse MS models (Zappia et al., 2005; Shimojima et al., 2016). It has also been demonstrated that the MSC secretome promotes ODC development, which improves remyelination and improves the functional state of mice induced with EAE (Bai et al., 2012). According to recent studies, EVs play a crucial role in the therapeutic benefits of MSCs and their secretome (Kråkenes et al., 2024).

#### 4.4.1 Exosomes

Recent studies have shown that exosomes originating from MSCs are significantly involved in the physiological activities of MSCs and may potentially yield more beneficial therapeutic outcomes compared to the original MSCs. Exosomes are a heterogeneous class of bilayer lipid membrane vesicles with a nano-sized diameter released by various cells consisting of adult MSCs. Initially, they are formed by endosomal membrane intraparticles to generate multivesicular bodies. Respecting molecular reports, exosomes produced by MSCs include a variety of molecular components, including lipids, proteins, RNA, and DNA profiles (Kråkenes et al., 2024; Kang et al., 2020; Zhou B. et al., 2020; Li, 2020; Ha et al., 2020; Liang et al., 2020; Shen et al., 2021; Gurung et al., 2021; Tan et al., 2024). MSCs-derived exosomes surrounded by a lipid membrane, as we discussed previously, keep their contents and permit them to migrate in tissues and targeted cells. They can participate in the pleiotropic functions of their parent cells, which include improving tissue regeneration. Currently, seven methods are available for effective exosome isolation, including differential centrifugation, ultrafiltration, flushing separation, mass spectrometry (MS), antibody affinity capture, precipitation, and microfluidic separation (Tang et al., 2021; Li et al., 2016; Zhang et al., 2018; Gao et al., 2022). Ultracentrifugation is frequently used to isolate exosomes. This method is not appropriate for isolating uncontaminated exosomes. Besides, immunoaffinity chromatography is a valuable method for obtaining pure exosomes. However, it is possible to approach this procedure by loading a small sample (Maqsood et al., 2020; Guan et al., 2020). The Tetraspanin family, which includes several proteins including CD9, CD63, and CD81, and some heat-shock proteins like Hsp90, Hsp70, and Hsp60, is abundant in the membrane. They act as markers and remain on the surface of the exosome. Importantly, exosomes produced by MSCs from younger or older hosts displayed various miRNA expression patterns (Fang et al., 2019).

Exosome biosynthesis begins with endosomal maturation, which entails specific changes to the endosomal membrane (Cunha e Rocha et al., 2024). Throughout this phase, the invagination process generates intraluminal vesicles (ILVs), leading to the formation of multivesicular bodies (MVBs). These MVBs can be transported to the plasma membrane for exocytosis, releasing ILVs as exosomes into the extracellular environment, or they can be directed to lysosomes for degradation. The specific mechanisms that determine whether exosomes evade degradation remain unclear. The three primary steps in exosome biosynthesis are cargo sorting, MVB transport and fusion with the plasma membrane, and MVB production (Cunha e Rocha et al., 2024; Kalluri et al., 2020).

It has strongly been evidenced that MSCs-derived exosome shows several merits such as neuroprotective effects, inherent stem cell source features, and BBB-crossing potential. However, exosomes may be effective drug delivery systems for neurodegenerative disorders therapy. They hinder local and systemic inflammation and have excellent biocompatibility, minimal immunogenicity, and low toxicity (Hosseini Shamili et al., 2019; Kyurkchiev et al., 2014). Recently, Fathollahi et al. administered MSC-derived exosomes to EAE mice via the intranasal (IN) route. The results demonstrated a considerable decrease in clinical scores associated with increases in immunomodulatory reactions, such as an increase in the percentage of CD25^+^ Foxp3+ Tregs and TGF-β levels (Fathollahi et al., 2021). In another study, placenta-derived MSCs (PMSCs)-exosome improved motor function in treated EAE mice more efficiently than PMSCs therapy. PMSC-exosome also decreased the damage of DNA in oligodendroglia and enhanced myelination in the treated mice’s spinal cord by stimulating endogenous ODC progenitor cells to develop into mature myelinating ODCs. Thereby, PMSC-derived EVs provide a practical option for cellular-based treatments for MS, as shown in the mice model of the disease (Clark et al., 2019). Jafarinia and his coworkers also studied and compared the effects of hADSC and hADSC-exosome on EAE in mice. Based on the results, the myelin ODC glycoprotein-induced splenocyte proliferation and the highest mean clinical score in hADSC and hADSC-exosome-treated animals were considerably lower than in control mice. The inflammation level and demyelination rates were also decreased following the administration of both hADSC-exosome and parental hADSC (Jafarinia et al., 2020). A recent study also showed that BM-MSCs cross the BBB and target neural cells. They could significantly enhance the numbers of newly generated ODCs and the level of MBP; moreover, BM-MSCs-exosome decreased neuroinflammation by enhancing the macrophage M2/M1 ratio and suppressing inflammatory TLR2/IRAK1/NFκB pathway (Zhang et al., 2022; Son et al., 2006; Ullah et al., 2015).

As the primary immune cells in the central nervous system, microglia are essential to the pathophysiology of MS because they promote both neuroinflammation and neurodegeneration. Microglia in MS adopt a pro-inflammatory M1 phenotype in response to CNS damage and inflammation, releasing cytokines such as TNF-α, IL-1β, and IL-6 (Liu et al., 2023; Zhang X. et al., 2023). MSC-derived exosomes contain therapeutic molecules that show promise in regulating microglial activation. Research has demonstrated that MSC exosomes can induce the polarization of microglia from the pro-inflammatory M1 phenotype to the anti-inflammatory M2 state. This transition is characterized by a decrease in the expression of pro-inflammatory cytokines and an increase in the production of anti-inflammatory cytokines, such as TGF-β and IL-10 (Liu W. et al., 2020). Furthermore, the phagocytic function of microglia is essential for removing myelin debris and apoptotic cells, which hinders remyelination. In MS, successful remyelination and neuronal survival rely on this process of elimination (Kråkenes et al., 2024). Specific miRNAs transported by MSC exosomes play a crucial role in regulating microglial polarization. Exosomes derived from hypoxic BM-MSCs have been shown to overexpress miR-216a-5p, which can reverse the release of inflammatory factors by microglia, including TNF-α, IL-6, and inducible nitric oxide synthase (Liu W. et al., 2020). Furthermore, research has demonstrated that miR-146a-5p and miR-125a reduce pro-inflammatory microglial activity following CNS damage. By inhibiting the TLR4/NF-κB/PI3K/AKT inflammatory cascade, MSC exosomes modify the inflammatory phenotype of microglia, shifting it towards an anti-inflammatory state (Liu W. et al., 2020; Zhang et al., 2021). Exosomes were administered intravenously as a single dose following spinal cord injury (SCI) in a mouse model to demonstrate this effect. Consequently, the mice that received exosomes exhibited significantly better performance compared to the control group (Liu W. et al., 2020).

In summary, MSC-derived exosomes represent a promising therapeutic avenue for MS and other neurodegenerative disorders. They offer multifaceted benefits through their immunomodulatory, neuroprotective, and regenerative properties, thereby paving the way for innovative and targeted treatment strategies.

## 5 Clinical trials

Autoinflammatory and autoimmune conditions are commonly managed with immunosuppressive medications, although their efficacy may vary among a diverse patient cohort. Consistent use of drugs may exacerbate adverse reactions, while prolonged suppression of the immune system heightens susceptibility to infections over time (Jung and Kim, 2022; Wigerblad and Kaplan, 2023). Recent studies have shown that MSCs are significantly involved in immune system regulation and tissue regeneration, suggesting their potential as a therapeutic approach for autoimmune conditions (Ding et al., 2015; Lv et al., 2014). Numerous recent clinical trials have been carried out using MSCs to manage MS. In the second phase of a randomized clinical trial, five patients with RRMS received MSC treatment for 6 months, leading to decreased brain MRI lesions (Llufriu et al., 2014b). Recently, Meng et al. (Meng et al., 2018) found that the systemic delivery of allogeneic UC-MSCs resulted in amelioration of the clinical manifestations in patients with MS. UC-MSCs therapy also reduced and Expanded Disability Status Scale (EDSS) and the frequency of foci, as determined with MRI. The most frequently reported adverse outcomes included elevated body temperature, head pain, and lightheadedness of feelings. The intervention also decreased levels of IL-2, CD86, HLADRB1, and CTLA-4 in peripheral blood (Meng et al., 2018). Other open-label prospective clinical trials (phase I/IIa) also revealed the clinical potentials of BM-MSCs in MS patients. Treatment reduced EDSS without altering lesion volume. Early-stage lesion reduction correlated with increased VEGF, IL-6, and IL-8 levels (Dahbour et al., 2017). Another study on 24 patients with active-progressive MS exhibited that the reduction in EDSS has an intimate association with increased FoxP3+CD4^+^CD25^+^ cells and decreased lymphocyte proliferation (Petrou et al., 2021a). Additional double-masked phase II clinical trials that were randomized and evaluated the effects of intrathecal (IT) or IV transplantation of MSC yielded comparable findings. The levels of NF-L CSF were notably reduced 6 months following the administration of MSC-IT treatment. Nine out of fifteen patients in the MSC-IT group experienced a reduction of more than 50% in their NF-L levels, as opposed to 33% in the MSC-IV group and 6.6% in the control group (Petrou et al., 2022). Llufriu et al. (2014b) found a non-significant decrease in the occurrence of Th1 (CD4^+^ IFN-γ+) cells in the bloodstream of patients who received autologous BMMSCs therapy. Of course, individuals who received MSC treatment demonstrated a reduced average total count of gadolinium-enhancing lesions (GEL). Finally, a shift from Th1 to Th2 immunity in hUCMSC-treated MS has been supported, according to reports (Li et al., 2014). On the other hand, Fernández et al. (2018) discovered that the IV administration of AT-MSCs did not lead to a statistically significant improvement in clinical outcome measures. These metrics encompassed the frequency of relapses, the EDSS score, the non-normalized cerebral volume on MRI scans, or the number of active lesions observed in gadolinium-enhanced T1 scans (Fernández et al., 2018). Yamout et al. (2010) administered autologous BM-derived MSCs via injection to nine with SPMS and one patient with RRMS. They documented improved clinical results in their patients following 3 months to 1 year. In this phase 2a clinical study, 10 individuals diagnosed with SPMS were administered MSCs intravascularly over 6 months. Following this intervention, the investigators examined to assess the impact of MSCs on the processes of remyelination and neuroprotection (Yamout et al., 2010). In a clinical study involving 15 patients with RRMS who had not responded to traditional disease-modifying therapies (DMTs), MSCs demonstrated systemic benefits for the immune system. The percentage of activated myeloid DCs and lymphocytes decreased while the number of regulatory T cells increased. Notably, these MSC-induced effects persisted *in vitro*, as immune cells from treated individuals demonstrated a reduction in lymphocyte proliferation. The effectiveness of MSC treatment was clinically supported by a reduction in the mean EDSS scores and the absence of new MRI lesions at the six-month follow-up (Karussis et al., 2010). Furthermore, in their 2007 study, M. Bonab and colleagues (MOHY et al., 2007) investigated the progression of the disease following the IT administration of MSC to 10 MS patients. Consequently, it has been observed that the advancement of the disease has progressively decelerated in 50% of the subjects being investigated (MOHY et al., 2007). Table 2 summarizes studies on the clinical application of stem cell therapy for MS and related adverse effects, along with observed results in patients.

**TABLE 2:** Therapeutic applications of MSCs and their utilization in clinical trials for the treatment of MS.

study	Study phase/Participant number	Cell source	Amount, Route, and time of appl.	Adverse effects	Results
Llufriu et al. (2014a)	Phase II/9 patients (5 MSCs, four placebo)	BM -MSCs	1–2 × 10⁶ MSCs/kg, IV injections, crossover design with 6-month follow-up per treatment arm	No serious adverse events	-There is a discernible trend indicating a reduction in the number of gadolinium-enhancing lesions (GELs) observed through MRI. The group receiving MSC treatment exhibited a statistically significant decrease in the mean cumulative GEL count. However, no notable effects were observed on the secondary endpoints
Petrou et al. (2022)	Phase II/48 patients (15 MSC-IT, 15 MSC-IV, 15 placebo)	MSC	IT or IV, single injection, evaluated at 6 months post-treatment	No serious adverse events	-Significant reduction in NF-L levels in the MSC-IT group compared to baseline and placebo; 9/15 patients in the MSC-IT group showed >50% reduction in NF-L. CXCL13 reduction was not statistically significant.
Yamout et al. (2010)	Pilot study/10 patients	Autologous BM -MSCs	IT injection, single dose, assessed at 3, 6, and 12 months	No serious adverse events	-EDSS improvement in 5/7 patients, stabilization in 1/7, and worsening in 1/7 at 3–6 months; MRI showed new lesions in 5/7 and Gadolinium (Gd+) lesions in 3/7 patients. Vision improvement in 5/6 patients
Li et al. (2014)	Not explicitly stated/23 patients (13 treated, 10 control)	hUC-MSCs	IV infusion of hUC-MSCs, three times in a 6-week period, combined with anti-inflammatory treatment	Not reported	-EDSS scores and relapse occurrence were significantly lower in hUC-MSC-treated patients compared to the control group. Symptoms improved. Shift from Th1 to Th2 immunity observed
Karussis et al. (2010)	Phase 1/2/34 patients (15 MS, 19 ALS)	Autologous MSCs	Mean of 63.2 × 10⁶ MSCs injected intrathecally (n = 34) and IV (n = 14); follow-up ≤25 months	Injection-related transient fever (21 patients); headaches (15 patients); no major adverse effects reported during follow-up	-Mean EDSS score improved from 6.7 to 5.9 in MS patients; ALSFRS score stable for 6 months
					-Immunomodulatory effects observed, including increased regulatory T cells and reduced lymphocyte proliferation.
Uccelli et al. (2021)	Phase 2/144 patients (69 early-MSC group, 75 placebo-first group)	Autologous BM -MSCs	Single IV dose; MSCs given at baseline for early-MSC group or at week 24 for delayed-MSC group; follow-up to week 48	213 adverse events recorded; infections and infestations most frequent (25%); no serious adverse events in the MSC group; no deaths reported	-MSC treatment was safe and well-tolerated but showed no significant effect on the total number of gadolinium-enhancing lesions (RR 0.94, p = 0.78). Further studies are needed to evaluate tissue repair effects
Tremblay et al. (2022)	Phase II/20 patients (9 early-treatment group, 11 delayed-treatment group)	Autologous MSCs	Single IV infusion; administered at Week 0 for the early-treatment group and at Week 24 for the delayed-treatment group	No significant adverse effects were reported	-No improvement in neurophysiological measures (corticomotor excitability or inhibition). Prolonged latency of motor evoked potentials and central motor conduction time. The decline in hand dexterity post-infusion
Berard et al. (2022)	Phase II/28 participants	MSCs	Not specified, likely IV infusion or other route; administered at Week 0, Week 24, and Week 48	No lasting adverse effects; some temporary cognitive decline observed at Week 24	-No detectable effect on cognition in the short term. Some cognitive stability or improvement was observed. There was a temporary decline in some cognitive regions at Week 24, with performance returning to baseline levels at Week 48. No lasting negative impact on cognition
Cohen et al. (2023)	Phase II/18 participants	Autologous MSCs (NurOwn^®^)	IT injection, three treatments administered (specific timing not provided)	Two participants developed low back and leg pain, consistent with arachnoiditis, after one of the IT treatments	-At 28 weeks, 19% of participants showed a ≥25% improvement over baseline in the timed 25-foot walk speed/nine-hole peg test. Consistent efficacy signs in other outcomes, a decrease in inflammatory biomarkers, and an increase in CSF neuroprotective factors
Harris et al. (2024)	Phase II/54 participants (27 MSC-NP, 27 placebo)	Autologous MSCs	IT injection, six injections (up to 10 million cells per injection), spaced 2 months apart (Year 1)	No significant adverse events. Prophylactic IV antibiotics and acetaminophen were used for some discomfort related to lumbar puncture	-No difference in EDSS Plus improvement between MSC-NP (33%) and placebo (37%) groups
					-Significant improvement in T25FW and 6MWT in patients with EDSS 6.0–6.5
					-Bladder function improved, and reduced grey matter atrophy
					-Biomarker analysis showed MMP9 increase and CCL2 decrease
Meng et al. (2018)	—	Umbilical cords	1 to 2 × 10^6^ cells/kg, IV, 3-month	Fever was the most common adverse reaction, followed by dizziness, headache, skin redness, and vascular irritation	-The patient’s unstable walking, coordination, appetite, mental state, balance, and numbness in the right limbs, as well as constipation, have improved
					-↓Expression of CTLA-4, IL-2, CD86, and IL-17c, TGF-β2, and HLA-DRB1, Foxp3
					-The lesions on the left side were significantly reduced on the MRI, and the volumes of the frontal and parietal lobes, as well as the semioval area and spinal cord, were decreased
Petrou et al. (2020)	I/II/48	BM	IT, 1 × 10^6^ IV, 1 × 10^6^, 14 months	Relapse of MS	-The improvements observed in optical coherence tomography (OCT) and motor networks are supported by findings from functional magnetic resonance imaging (fMRI)
Harris et al. (2018)	I/20	MSC-derived Neural Progenitors (MSC-NPs)	IT, 1 × 10^7^,3-month	Headaches and fever	-Urodynamic improvement in bladder function post-treatment improvement in T25FW speed decrease in median EDSS
Fernández et al. (2018)	I/II/30	ADMSCs	IV,1 × 10^6^ cells/kg or 4 × 10^6^ cells/kg, 3-month	Urinary infection, respiratory infection, and anemia	-Baseline MRI data had no significant differences
Harris et al. (2021)	I/20 patients	MSC-NPs from BM	IT, 9.4 × 10^6^, 30-month	Minor headache	-Subjects who received multiple injections of IT-MSC-NP showed either a reversal in disability or a lack of disease progression
					-↓ CCL2, ↑ TGF-β2
					-EDSS and T25FW showed improvement
					-The MRI scans of the brain did not reveal any alterations
Riordan et al. (2018)	—	UCMSC	20 × 10^6^, IV, over 7 days	No serious adverse events	-The administration of IV infusions of UCMSC to individuals with MS is safe
Dahbour et al. (2017)	I/IIa/15	Autologous BM -MSCs	110 × 10^6^ cells,	Localized pain and headache	-Numerous patients reported improved visual clarity
			IT, 1-month interval		-Increase in IL-8, IL-6, MCP-1 & VEGF
					-T-25-FWT showed an overall improvement trend
					-MMSE: no change
					-9-PH showed a trend of improved
					-The lesion volume, which increased significantly in MRI
Fernández et al. (2018)	I/II	Adipose-derived MSCs (AdMSC)- Abdominal subcutaneous	Three groups: placebo, low-dose (1 × 10^6^ cells/kg) or high-dose (4 × 10^6^ cells/kg), Intravenously, patients with SPMS,	Urinary infection, respiratory infection, and anemia	-No significant changes from baseline in Bun, Cr, chol, Vital signs, spirometry
				None of these severe AEs	-Non-statistically significant differences between placebo and treatment groups in visual evoked potential (VEP) and SEP
					-There were no significant changes in CSF, OCT measurements, cognition, or quality of life questions
					-The mean EDSS score did not show statistically significant variations,
					-No change in low or high-dose groups MRI
Petrou et al. (2021b)	—	Autologous BMMSCs in 24 patients with active-progressive MS	The patients received an initial treatment of 1 ×10^6^ MSCS/kg of body weight (administered IT and IV), followed by up to eight additional courses of MSCs at intervals of 6–12 months	Headache. Low-grade fever, backache	-Multiple sessions of MSC therapy in individuals with progressive MS were found to be safe in the short to intermediate term. The treatment also resulted in clinical improvements, particularly in patients who received more than two injections, which were sustained for up to 4 years. These improvements were accompanied by short-term immunomodulatory effects

## 6 Integration of disease-modifying therapies and cell therapy in MS treatment

There are presently specific disease-modifying treatments (DMTs) available to stop the accumulation of structural brain damage associated with MS and its adverse effects on MS patients (Filippi et al., 2022; Wiendl et al., 2021). The advent of more effective DMTs during the past several years has significantly changed the landscape of MS treatment (Comi et al., 2017; Giovannoni et al., 2020; Goldschmidt and McGinley, 2021). Currently, available DMTs are categorized based on their efficacy into two primary classifications: high-efficacy (HE) DMTs and moderate-efficacy (ME) DMTs. The HE DMTs include natalizumab, fingolimod, ozanimod, siponimod, alemtuzumab, cladribine, ocrelizumab, and ofatumumab. In contrast, the ME DMTs comprise glatiramer acetate, interferon-beta (IFN-β), teriflunomide, and dimethyl fumarate (Comi et al., 2017; Giovannoni et al., 2020; Simpson-Yap et al., 2021). Additionally, high-dose methylprednisolone is frequently used to manage acute relapses by suppressing inflammation (Sormani et al., 2021; Travers et al., 2022). Subcutaneous IFN-β1b, the first MS DMT ever created, was authorized by the Food and Drug Administration (FDA) in 1993 to treat progressive relapsing MS (PRMS) (Bayas and Gold, 2003).

DMTs have demonstrated effectiveness in managing MS; however, they are associated with several limitations. These limitations include heterogeneous responses among patients, the potential for long-term toxicity, and an incomplete capacity to halt disease progression, particularly in the later stages of the condition (Langer-Gould et al., 2023). In contrast, cell-based therapies, particularly those utilizing MSCs, have garnered attention due to their anti-apoptotic properties, paracrine signaling capabilities, and multidirectional differentiation potential. These characteristics have prompted their investigation in translational research and clinical trials aimed at addressing prevalent diseases, including neurological disorders that affect CNS structures, such as stroke, Huntington’s disease (HD), Parkinson’s disease (PD), MS, and SCI (Andrzejewska et al., 2021).

### 6.1 Advantages of combined approaches

• Synergistic effects: DMTs reduce systemic inflammation and immune activation, potentially creating an environment that fosters MSC-mediated repair and neuroprotection. For example, the immunomodulatory effects of IFN-β or natalizumab may enhance the anti-inflammatory cytokines secreted by MSCs (Dadfar et al., 2024; Gharibi et al., 2015; Emamnejad et al., 2019).
• Broad therapeutic coverage: While DMTs primarily target immune dysregulation, MSCs directly address neurodegeneration and promote remyelination, thereby tackling different aspects of the disease’s pathophysiology (Orrù et al., 2024; Karussis et al., 2008).
• Enhanced Efficacy: Combining cell therapy and DMTs may reduce relapse rates more effectively than either treatment alone. More importantly, this combination may also accelerate recovery from damage (Peterson et al., 2022).

### 6.2 Challenges and limitations

The development of novel DMTs has advanced significantly in recent years, particularly over the past decade. However, much work remains to be done before a broader range of alternatives becomes available to MS patients with varying clinical presentations. Only a few treatments have been thoroughly researched for more severe and active forms of MS, such as SPMS and PPMS. Consequently, many patients continue to experience substantial disease progression despite current DMT therapies. Additionally, there is a considerable risk of adverse effects associated with existing DMTs, including infusion reactions, infections, liver toxicity, and cardiovascular complications (McGinley et al., 2021). These medications, particularly natalizumab and fingolimod, have the potential to cause progressive multifocal leukoencephalopathy (PML), a disease associated with a high fatality rate (Sriwastava et al., 2021). In addition, many DMTs are incredibly costly. The lifetime direct medical expenses for a patient with MS are estimated to be $4.8 million, making it the second most expensive chronic medical condition after heart failure. Furthermore, DMTs remain the single most significant contributor to these costs. In 2020, the median annual cost of available DMTs was $91,835, with several therapies exceeding this amount. Therefore, to reduce costs, it is crucial to keep looking at less expensive options for efficacy potential and to keep diversifying therapies (Hartung, 2021).

Understanding how DMTs influence the homing, engraftment, and therapeutic efficacy of MSCs remains an area of ongoing research. For instance, fingolimod alters lymphocyte trafficking, which might interact with MSC migration dynamics (Kassis et al., 2021; Wiendl et al., 2021; Yazdi et al., 2018). Clinical trials evaluating the safety and efficacy of such combinations will be critical for developing protocols that maximize patient outcomes while minimizing adverse effects.

## 7 Follow-up and evolutionary biomarkers after MSC administration

An essential component of MSC therapy involves monitoring the therapeutic outcomes and identifying potential complications through follow-up and evolutionary biomarkers. These biomarkers provide crucial insights into the dynamics of MSC behavior, their interaction with the host environment, and the overall therapeutic efficacy (Ghareghani et al., 2024; Iacobaeus et al., 2019; Granchi et al., 2019). MSCs exert immunomodulatory effects, which can be tracked using biomarkers such as IL-4, IL-10, IL-13, IL-16, and TGF-β, and reductions in pro-inflammatory cytokines like IL-6 and TNF-α. The normalization of these markers indicates the anti-inflammatory efficacy of MSCs, particularly in diseases like MS, where inflammation is a hallmark (Petrou et al., 2022; Li et al., 2014). Neurofilament proteins (NF), which are released into the CSF following axonal injury in the central nervous system, serve as reliable indicators of axonal damage and neuronal death. Among these, the neurofilament light chains (NF-L) are the most extensively studied subtype (Cairns et al., 2004). Since NF are essential parts of the neuron’s cytoskeleton, any neurological condition that damages neurons or axons may result in elevated CSF levels of these proteins. The CSF of patients with MS consistently contains elevated levels of NF-L, indicating that NF-L may function as a biomarker for MS disease activity, including subclinical activity, as well as for the responsiveness to various MS therapies. Additionally, studies have demonstrated that increased blood levels of NF-L in the early stages of MS may predict future increases in MS lesions and brain atrophy (Williams et al., 2021; Chitnis et al., 2018; Novakova et al., 2017; Håkansson et al., 2017). The most potent B-cell chemoattractant, the CXCR5 ligand CXCL13, is present in both active lesions of MS and the CSF of MS patients. Elevated levels of CXCL13 have been shown to predict the progression from clinically isolated syndrome (CIS) to MS. Furthermore, research indicates that CXCL13 is associated with disease exacerbations and a poorer prognosis in MS (Khademi et al., 2011). MSC-mediated healing processes can be identified by tissue regeneration markers such as matrix metalloproteinases (MMPs), VEGF, and fibroblast growth factor (FGF). Particularly in the context of MS, these indicators are valuable for assessing the recovery of vascular and neuronal structures in the central nervous system (Gavasso et al., 2024; Hofer and Tuan, 2016; Farooq et al., 2021).

Biomarkers like C-reactive protein (CRP) and systemic metabolic markers, such as changes in glucose and lipid profiles, provide insights into broader systemic responses to MSC administration (Yang et al., 2018; Weiss et al., 2021).

A class of micromolecules with potential as biomarkers for MS is microRNAs, which are small non-coding RNAs that regulate post-transcriptional gene expression (Raphael et al., 2015).

Research on specific metabolic pathways associated with the pathophysiology of MS provides an additional approach to identifying biomarkers. For example, studies have shown that the kynurenine pathway, the primary mechanism for tryptophan degradation, regulates immune activity. Evidence suggests that during relapses, the CSF of MS patients exhibits elevated levels of the neuroprotective metabolite kynurenine acid (Lim et al., 2010).

By integrating these biomarkers into the clinical evaluation framework, it becomes possible to optimize the therapeutic potential of MSCs while minimizing adverse effects.

## 8 Challenge of MSC therapy in MS

In recent years, there has been a significant focus on stem cell therapy. MSC therapy in translational medicine has considerable expectations. However, various aspects of MSC treatment need to be well-defined. Given the variety of methods explored, the full extent of the potential impacts of MSC therapy remains uncertain (Lukomska et al., 2019). Moreover, due to their possible application in autologous transplantation, MSCs have gained significant clinical interest. Numerous clinical trials involving MSCs have been conducted, with many more currently under investigation. As the clinical use of MSCs continues to expand, particularly in the context of both autologous and allogeneic transplantation, long-term monitoring of patients is essential to assess the safety and efficacy of MSC therapy. According to recent studies, thousands of patients have received culture-expanded allogeneic or autologous MSCs to treat various diseases (Squillaro et al., 2016; Shandil et al., 2022). MSC treatment has proven highly effective in most cases; however, long-term monitoring remains crucial to assess the potential hazards associated with MSC transplantation. Numerous *in vitro* and *in vivo* investigations provided evidence for MSC differentiation into specific cell types (Nowakowski et al., 2016). While most *in vivo* studies have confirmed the safety of MSC therapy and demonstrated promising results, the therapeutic benefits of MSC-based treatments remain limited. Furthermore, there are potential risks associated with using MSCs in specific cellular niches that should be carefully evaluated in long-term follow-up studies (Lukomska et al., 2019).

Although MSC therapy holds significance in treating MS and other diseases, it is essential to acknowledge the potential adverse effects associated with its administration. One of the most notable challenges is the method of administration. The route of administration significantly influences the therapeutic outcome, and it has been shown that different routes can lead to varying levels of efficacy and safety (Mansoor et al., 2019; Lukomska et al., 2019). The approach to administering MSCs is greatly determined by the specific therapeutic objectives. For instance, IV administration, one of the most common routes, has been associated with limited success in MS models. Studies indicate that MSCs administered IV are often trapped in the lungs and liver, and their presence in the inflammatory lesions of the CNS is minimal. This inefficiency in homing to the target tissue is primarily due to the BBB, which prevents the passage of MSCs into the brain, thus limiting their therapeutic effects in MS patients (Abramowski et al., 2016; Cerri et al., 2015). Moreover, MSCs derived from BM have shown poor therapeutic efficacy when administered systemically. They fail to reach the damaged neurons, and in some cases, they are cleared from the system within a month post-administration (Neirinckx et al., 2021). The use of local administration techniques is restricted due to the associated risks of direct tissue injection or intraventricular infusion aimed at enhancing MSC homing. While local injections have the potential to deliver drugs precisely where they are needed, they also carry the risk of varying degrees of local inflammation, infection, or tissue damage at the injection site. The dosage and frequency of MSC therapy directly influence both safety and effectiveness. Excessive administration of MSCs may lead to abnormal tissue growth due to overuse, immune reactions or tumor formation resulting from improper dosing. It is crucial to understand how the doses are spatiotemporally related and to determine the optimal total number of doses to minimize cumulative adverse effects over time (Caplan et al., 2019; Galipeau and Sensébé, 2018; Kabat et al., 2020; Afkhami et al., 2024). Regarding broader systemic effects, adverse reactions can vary from transient symptoms, such as nausea, fever, and headache, to more serious complications. Several studies have documented these adverse effects, including the occurrence of transient symptoms like vomiting, nausea, and impaired visual acuity in 4.3% of individuals receiving MSC infusion for steroid-resistant graft-versus-host disease (GVHD) (Table 3) (Dotoli et al., 2017). These side effects underscore the importance of monitoring patients during and after MSC treatment, especially when high doses or repeated infusions are involved.

**TABLE 3:** The advantages and disadvantages of therapies based on MSCs.

Different sources of MSCs	Advantages	Disadvantages
BM-MSCs	-Support hematopoiesis through the formation of hematopoietic-supporting colonies. Differentiate into mesodermal lineage cells (Uccelli et al., 2011)	-Painful and invasive extraction methods with limited efficiency (Quirici et al., 2002)
	-Release BDNF and promote ODC generation	-Ineffectiveness in managing stabilized MS progression (Darlington et al., 2011)
	-Show immunomodulatory effects in the early stages of neurodegenerative diseases like MS (Darlington et al., 2011)	-Risk of malignant transformation and immune rejection after clinical use (Røsland et al., 2009; Huang et al., 2010)
UCMSCs	-Minimally invasive extraction methods with fewer ethical concerns. Rapid cell division capacity and low immunogenicity. Differentiate into multiple cell lineages (Ding et al., 2013)	-Potential for tumor formation and rejection by the immune system following clinical application (Herberts et al., 2011)
	-Produce neurotrophic factors and exhibit immunomodulatory effects in autoimmune disease models (Musiał-Wysocka et al., 2019; Dong et al., 2018)	-The coagulation-promoting properties may potentially play a role in the formation of pulmonary embolism (Tatsumi et al., 2013)
		-The potential for viral and prion transmission following administration poses a significant risk (Mazini et al., 2019; Chen et al., 2014)
AD-MSCs	- Convenient and efficient isolation process. Ability to differentiate into various lineages (e.g., adipogenesis, neurogenesis, cardiogenesis, chondrogenesis, myogenesis, osteogenesis) (Mazini et al., 2019)	-Potential for tumorigenicity and immune rejection in clinical applications (Kim et al., 2018)
	-Migration to various organs by activating α4 integrin expression (Kashani et al., 2012)	-Risk of nephrotoxicity and procoagulant properties (Musiał-Wysocka et al., 2019; Tatsumi et al., 2013; Kim et al., 2018)
	-Generate multiple growth factors beneficial for therapy (Dai et al., 2016)	

Lastly, the quality of the MSC product and its source play an essential role in determining safety outcomes. The donor’s age appears to be the most critical parameter to consider. MSCs derived from older donors or patients with comorbidities may exhibit compromised functionality, affecting their therapeutic potential and increasing the risk of adverse reactions. This challenge is especially relevant in autologous transplantation, where geriatric patients may struggle to obtain a sufficient number of viable MSCs for treatment (Dufrane, 2017; Liu et al., 2017; Kokai et al., 2017; Pachón-Peña et al., 2016).

In summary, while MSCs present a novel and promising approach to treating MS, it is crucial to recognize the potential adverse effects and complications that may arise from their application.

## 9 Conclusion

MSC therapy has demonstrated significant potential as an innovative approach for managing MS, addressing both the immunological and neurodegenerative aspects of the disease. MSCs exhibit robust immunomodulatory properties, promote remyelination, and support neuroregeneration, making them a promising candidate for comprehensive MS therapy. Preclinical and clinical studies have shown encouraging results, particularly in reducing inflammation and slowing disease progression. However, limitations such as optimal dosing, delivery methods, and long-term safety concerns remain critical challenges. Despite these challenges, MSC therapy represents a transformative step forward in personalized medicine for MS, offering hope for improved quality of life for patients. Future research should address the remaining challenges associated with MSC therapy, such as optimizing delivery routes and dosing regimens to enhance therapeutic efficacy and reduce potential adverse effects.
